# Circulating plasma and exosome levels of the miR-320 family as a non-invasive biomarker for methamphetamine use disorder

**DOI:** 10.3389/fpsyt.2023.1160341

**Published:** 2023-04-25

**Authors:** Wenjin Xu, Qingxiao Hong, Yun Zhou, Xiaoyu Chen, Longhui Li, Majie Wang, Weisheng Chen, Xiaohu Xie, Dingding Zhuang, Miaojun Lai, Wenhua Zhou, Huifen Liu

**Affiliations:** ^1^Laboratory of Behavioral Neuroscience, Ningbo Kangning Hospital, Ningbo Institute of Microcirculation and Henbane, School of Medicine, Ningbo University, Ningbo, Zhejiang, China; ^2^Key Laboratory of Addiction Research of Zhejiang Province, Ningbo, Zhejiang, China

**Keywords:** exosome, plasma, methamphetamine, microRNAs, diagnosis, biomarker

## Abstract

The neurobiological mechanism underlying methamphetamine (MA) use disorder was still unclear, and no specific biomarker exists for clinical diagnosis of this disorder. Recent studies have demonstrated that microRNAs (miRNAs) are involved in the pathological process of MA addiction. The purpose of this study was to identify novel miRNAs for the diagnosis biomarkers of MA user disorder. First, members of the miR-320 family, including miR-320a-3p, miR-320b, and miR-320c, were screened and analyzed in the circulating plasma and exosomes by microarray and sequencing. Secondly, plasma miR-320 was quantified by real-time quantitative reverse transcription polymerase chain reaction (RT-qPCR) in eighty-two MA patients and fifty age-gender-matched healthy controls. Meanwhile, we also analyzed exosomal miR-320 expression in thirty-nine MA patients and twenty-one age-matched healthy controls. Furthermore, the diagnostic power was evaluated using the area under the curve (AUC) of the receiver operating characteristic (ROC) curve. The expression of miR-320 significantly increased in plasma and exosomes of MA patients compared with healthy controls. The AUC of the ROC curves of miR-320 in plasma and exosomes of MA patients were 0.751 and 0.962, respectively. And the sensitivities of miR-320 were 0.900 and 0.846, respectively, whereas the specificities of miR-320 were 0.537 and 0.952, respectively, in plasma and exosomes in MA patients. And the increased plasma miR-320 was positively correlated with cigarette smoking, age of onset, and daily use of MA in MA patients. Finally, cardiovascular disease, synaptic plasticity, and neuroinflammation were predicted to be the target pathways related to miR-320. Taken together, our findings indicated that plasma and exosomal miR-320 might be used as a potential blood-based biomarker for diagnosing MA use disorder.

## Introduction

Exosomes, with an average diameter of around 100 nanometers, are a subset of extracellular vesicles (EVs). The formation of exosomes originates from endocytosis. Exosomes are multivesicular bodies formed by the invagination of lysosome particles in cells and released to extracellular vesicles after the fusion of multivesicular bodies in the outer membrane and cell membrane (1). Exosomes are components of EVs and widely exist in various body fluids, such as blood, cerebrospinal fluid, urine, and breast milk. Many types of cells could secrete exosomes under normal and pathological conditions, carrying a large number of bioactive cargoes such as nucleic acids, proteins, lipids, and metabolites from host cells, and playing a key regulatory role in intercellular communication (2). In addition, exosomes could regulate important physiological processes including cell differentiation, immune responses, and tissue homeostasis (2, 3).

Many studies focused on the biological activity of exosomes, especially microRNAs (miRNAs) contained in exosomes. miRNAs are non-coding single-stranded small RNA molecules with a length of about 22 nucleotides that widely exist in eukaryotic cells and tissues. miRNAs degrade the mRNA of target gene or inhibit the translation of target gene mRNA through complementary pairing with the 3’UTR (4). miRNAs could regulate neural plasticity, and modify synaptic structure, function and morphology. Therefore, miRNAs are considered to be closely related to cognitive functions such as learning and memory. Meanwhile, miRNAs is highly enriched in addiction-related brain circuits, suggesting that miRNAs might play an important role in drug addiction (5). Exosomal proteins and miRNAs are novel promising biomarkers in clinical diagnosis (6). Compared with biomarkers identified in conventional specimens such as blood, urine, or semen, exosomal miRNAs have the advantages of non-invasiveness, stability, and high specificity, Moreover, exosomes could cross the blood–brain barrier, evade the body’s immune response, and have few adverse reactions. Therefore, exosomal miRNAs have been well studied in neurodegenerative disorders and brain tumors (7–9).

According to 2021 Annual Report of the Drug Control in China, MA has replaced heroin to be the most popular illegal drug in China. The biological mechanisms underlying MA addiction have not been fully clarified. Clinical diagnoses are mainly based on subjective reports of Diagnostic and Statistical Manual of Mental Disorders, Fifth Edition (DSM-5) questionnaire. While, objective diagnostic indicators for MA addiction is still lacking. Therefore, it is of great clinical significance to explore non-invasive, objective, sensitive, and specific biomarkers for this disorder. The isolation of plasma exosomes and the analysis of their cargo may provide a novel diagnostic potential for diverse disorders (10). In addition, there are few miRNAs studies based on the plasma or exosomes from MA addiction patients. Therefore, it is important to investigate the role of miRNAs in plasma exosome of MA addiction, and to develop biomarkers for the diagnosis of MA use disorder.

In this study, we tested the hypothesis that MA abuse could change the expression of miR-320 in plasma and exosomes. We collected the circulating plasma and clinical features (gender, age, daily dose of MA, etc.) of objectives, and verified the expression of miR-320 in plasma and exosomes. In addition, we explored the correlation between miR-320 and clinical characteristics. Receiver operating characteristic (ROC) curve analysis was used to assess the diagnostic power of miR-320 for MA use disorder.

## Materials and methods

### Study participants and ethical statement

We enrolled patients with MA addiction in the compulsory isolation drug rehabilitation center in Ningbo before detoxification treatment, and we also enrolled age-and gender-matched healthy controls from Ningbo blood bank between 2020 and 2021. All subjects were recruited according to the following criteria: (a) no major infectious diseases, other major chronic diseases, or family history of diseases; (b) informed consent given to participate this project; (c) patients with MA addiction were diagnosed using DSM-5 questionnaire, and urine, blood, or hair tests were positive. The exclusion criteria were as follows: (a) a current history of physical disease or history of infectious diseases; (b) inability to give informed consent; (c) abuse of another drug. All patients recorded their clinical data (including age, gender, and MA use information) through questionnaires. Ethics were approved by the ethics committee of Ningbo Kangning Hospital (No. NBKNYY-2020-LC-53). The study protocols and methods were reviewed and approved by Ningbo Kangning Hospital.

### Blood sample collection and preparation

Blood samples were collected from all participants when entering the centers: 10 ml blood collection tubes containing ethylenediamine tetra-acetic acid were used to collect 6 ml blood samples. After collection, tubes were stored at 4°C until centrifugation, and the separation of plasma was performed under 1,500 g centrifugation for 10 min within 4 h of collection. Subsequently, the plasma was transferred to a new RNase/DNase tube and stored at −80°C.

### Isolation of circulating exosomes

Exosomes were separated by ultracentrifugation. Briefly, the plasma sample was centrifuged at 10,000 g for 30 min to eliminate shed microvesicles. Then, the supernatant was collected and filtered with a 0.22 μm membrane filter, centrifuged at 10,0000 g for 2 h, and washed with 1 × PBS. Finally, the exosomes were resuspended in 1 × PBS and stored at −80°C until further use.

### RNA extraction

Total RNA was extracted from plasma or exosomes using the miRNeasy Serum/Plasma Kit (Qiagen GmbH, Hilden, Germany) or Trizol reagent (Thermo Fisher Scientific, Massachusetts, USA), according to the manufacturer’s protocol. Subsequently, RNA concentration and purity were detected using nanodrop 2000 apparatus (Thermo Fisher Scientific, Massachusetts, USA).

### miRNAs microarray for plasma

The total RNA of five patients with MA use disorder and five healthy controls was extracted from plasma for miRNAs microarray screening. The miRNAs expression was determined using the Human miRNAs Microarray V19.0 (Agilent Technologies, California, USA), which contains probes for a total of 2006 miRNAs. The NanoDrop-2000 (Thermo Fisher Scientific, Massachusetts, USA) was used to quantify total RNA. Additionally, an Agilent Bioanalyzer 2,100 system (Agilent Technologies, California, USA) was used to assess RNA integrity. miRNAs microarrays mainly determine the sample labeling, microarray hybridization, and washing steps. All operations were performed according to the manufacturer’s instructions. Briefly, total miRNAs were dephosphorylated and denatured, followed by Cy3 labeling. After purification, labeled RNAs were hybridized onto the microarray according to the array manual. After washing, the slides were scanned into image files with the Agilent Scanner G2565CA (Agilent Technologies), and the scanned images were analyzed using Feature Extraction software 10.7 (Agilent Technologies). Data were analyzed using Gene Spring Software 12.6 (Agilent Technologies). Differentially expressed miRNAs were identified by calculating the fold change (FC) and *p*-value using *t*-tests with Benjamini–Hochberg correction. FC > 2 and *p* < 0.05 were considered statistically significant.

### miRNAs sequencing for exosomes

The total RNA extracted from exosomes was inspected and quantified using agarose gel electrophoresis and the nanodrop-2000. An miRNAs sequencing library was constructed using the NEB Multiplex Small RNA Library Prep Set for Illumina, according to the manufacturer’s protocol. In brief, approximately 1 g total RNA was processed to generate a small RNA library using TruSeq Small RNA Sample Prep Kits (Illumina) according to the manufacturer’s instructions. Purified RNAs were ligated with 3′ and 5′ adapters. Reverse transcription followed by polymerase chain reaction was used to synthesize cDNA, and purified cDNA library products with size of 135–155 bp were subjected to single-end sequencing on an Illumina NextSeq 500 according to the manufacturer’s protocol. For each RNA library, 20 million reads (raw sequencing data) generated. The raw sequencing data were analyzed by cutadapt Software to remove adapter dimers, low complexity, junk and repeats. To identify known miRNAs and novel 3p- and 5p-derived miRNAs, unique sequences of length 18–26 nt were mapped to Homo sapiens precursors in miRBase 22.0. The quantitative expression of miRNAs was obtained by the miRDeep2 software through comparison results to known genomes and statistics. miRNAs with mean CPM (Counts per million reads) ≥ 1 in each group were used for statistical analysis, and edgeR was used for differential expression analysis of miRNAs between groups. Differentially expressed miRNAs were identified by calculating FC and *p*-value using *t*-tests. FC > 2 and *p* < 0.05 were considered statistically significant.

### Quantification of candidate miRNAs

Candidate miRNAs were detected using RT-qPCR. Complementary DNA was synthesized using miScript^®^II RT Kit (Qiagen GmbH, Hilden, Germany) according to the manufacturer’s protocol. A MiScript SYBR Green PCR kit (Qiagen GmbH, Hilden, Germany) was used for the quantitative detection of miR-320 in plasma. The sequences of miR-320 primers were as follows: forward: 5’-GGGGGAAAGCTGGGTTG-3′; reverse: 5’-GTGCGTGTCGTGGAGTCG-3′. All reactions were performed in triplicate and normalized to cel-miR-39 (5’-UCACCGGGUGUAAAUCAGCUUG-3′), which was spiked to normalize the plasma samples at the onset of RNA isolation (11). To determine miR-320 in exosomes, a total of 200 ng RNA from each sample was mixed with 1 l dNTPs, 2 μl 10x RT buffer, 0.3 μl specific primers (Table 1), 0.2 μl M-MuLV Reverse Transcriptase, and 0.3 μl RNase inhibitor, and the mixture was made up to 20 μl with DNase-and RNase-free water. For miR-320 detection in exosomes, 2 × PCR master mix was used for quantitative analysis. The reaction system was configured as follows: 2 μl 2 × Master mix, 1 μl primer, 2 μl cDNA template, and the mixture was made up to 10 μl with DNase-and RNase-free water. The primer sequence used for miR-320 was the same as for plasma. All reactions were performed in triplicates and normalized to hsa-miR-423-5p: forward: 5’-TGAGGGGCAGAGAGCGA-3′; reverse: 5’-GTGCGTGTCGTGGAGTCG-3′ (12). The relative expression of miRNA was calculated by using the 2^−△△Ct^ method.

**Table 1:** RT primers for cDNA synthesis.

miRNAs	RT primer
hsa-miR-423-5p	5’GTCGTATCCAGTGCGTGTCGTGGAGTCGGCAATTGCACTGGATACGACAAAGTC3’
hsa-miR-320a	5’GTCGTATCCAGTGCGTGTCGTGGAGTCGGCAATTGCACTGGATACGACTCGCCCT3’
hsa-miR-320b	5’GTCGTATCCAGTGCGTGTCGTGGAGTCGGCAATTGCACTGGATACGACTTGCCC3’
hsa-miR-320c	5’GTCGTATCCAGTGCGTGTCGTGGAGTCGGCAATTGCACTGGATACGACACCCTC3’

### Characterization and quantification of exosomes

The extracted exosomes were analyzed using transmission electron microscopy. First, exosomes were fixed with 2% paraformaldehyde. Then, the exosome suspension was added to the Formvar/carbon-loading copper grid, and the formvar membrane was allowed to absorb while drying for 20 min. The grids were washed with distilled water, fixed with 1% glutaraldehyde for 5 min, washed again, and contrasted with uranyl acetate for 5 min. Grids were examined using a Tecnai G2 Spirit 120 kV electron microscope. In addition, we used Western blot to detect the specific expressed proteins in the exosome (13). Briefly, bicinchoninic acid method was used to analyze the concentration of isolated exosome protein; 10% SDS-PAGE was used to separate the total protein and transfect into the polyvinylidene fluoride membrane. Then, Western blocking solution was added to block the membrane for 2 h, and TSG101 (Santa Cruz Biotechnology, SC-7964), CD81 (Abcam, Ab79559), and CD63 (Abcam, Ab9479) antibodies were incubated overnight at 4°C. The membrane was washed three times with Western washing solution. Then, HRP-labeled goat anti-rabbit IgG (Beyotime, A0208) secondary antibody was added to bind the primary antibody for 2 h at room temperature. Finally, the membrane was placed in ChemiScope 6,000 Pro (Clinx, Shanghai, China) for quantitative analysis, and the relative density value was calculated using GAPDH (Abcam, Ab181603) as the internal control.

### Statistical analyses

All data were analyzed using SPSS 16.0 statistical software (IBM, Armonk, USA). Measurement data are shown as the mean ± standard error mean (SEM); classification data are represented by the proportion or rate. A normality test was performed for continuous variables using a Kolmogorov–Smirnov test. For comparing two independent groups, continuous variables were analyzed by Student’s *t*-test to determine statistical significance, and categorical variables were analyzed by Chi-square test or Fisher’ exact test. Normally distributed variables were analyzed by Student’s *t*-test to determine statistical significance, and nonparametric data were assessed using a Mann–Whitney *U*-test. For comparing multiple groups, the statistical significance of normally distributed variables with unequal variances was assessed by Welch’s ANOVA test followed by Games-Howell post-hoc test for multiple comparisons, and abnormally distributed variables were determined by the Kruskal–Wallis test followed by Dunn’s multiple comparisons *post hoc* test. *Z* tests were used for data not conforming to normal distributions, classification data are represented by the proportion or rate, and classification data were assayed using the χ^2^ test or Fisher’s exact probability method. Pearson’s or Spearman’s rank correlations were used to determine the relationship between the expression of hsa-miR-320 and clinical characteristics. ROC curves were used to assess the diagnostic accuracy between MA addiction and healthy control. Logistic regression analysis was used to adjust for confounding factors and estimate the odds ratio (OR) of the expression of miR-320 and clinical characteristics. Graphs were generated using the GraphPad Prism software 8.0 (GraphPad, San Diego, USA). For all empirical tests, *p <* 0.05 was considered statistically significant. Significance difference was denoted as *p < 0.05, **p < 0.01, and ***p < 0.001, unless noted otherwise.

## Results

### Participant characteristics

The clinical characteristics of the participants were shown in Table 2. We employed 50 healthy controls and 82 MA patients for the study of plasma miRNAs. The average ages of healthy controls and MA patients were 43.48 ± 1.30 years and 36.58 ± 0.92 years, respectively. No significant differences were found between groups in terms of gender (*p* > 0.05). Age, marital status, education, employment differed significantly between two groups (*p* < 0.001). Among MA patients, 53 (64.63%) had little education (middle school or below). The mean age for first MA use was 29.59 ± 0.98 and the average MA use time was 52.10 ± 4.66 months for MA patients. The majority of MA patients snorted MA.

**Table 2:** Demographic characteristics of the participants.

Variables	Plasma		*t*/χ^2^	*p*	Exosome		*t*/χ^2^	*p*
	Control (*n* = 50)	MA use disorder (*n* = 82)			Control (*n* = 21)	MA use disorder (*n* = 39)		
Age (years)	43.48 ± 1.30	36.58 ± 0.92	4.29	<0.001	36.00 ± 1.38	30.52 ± 1.46	−0.13	0.9
Gender (%)								
Male	24 (48)	33 (40.25)	0.76	0.38	8 (38.10)	7 (17.95)	2.96	0.086
Female	26 (52)	49 (59.75)			13 (61.90)	32 (82.05)		
Marital status (%)								
Single	13 (26)	29 (35.37)	27.07	<0.001	5 (23.81)	16 (41.03)	10.94	0.006
Married	35 (70)	24 (29.27)			15 (71.43)	11 (28.21)		
Divorced	2 (4)	28 (34.15)			1 (4.76)	11 (28.21)		
Widowed	0 (0)	1 (1.21)			0 (0)	1 (2.55)		
Education (%)								
Primary and below	1 (2)	19 (23.17)	82.81	<0.001	1 (4.77)	5 (12.82)	26.52	< 0.001
Middle school	1 (2)	34 (41.46)			1 (4.77)	19 (48.72)		
High school	8 (16)	23 (28.05)			6 (28.58)	13 (33.33)		
College and above	40 (80)	6 (7.32)			13 (61.90)	2 (5.13)		
Employment (%)								
Yes	47 (94)	42 (51.22)	25.88	<0.001	20 (95.24)	22 (56.41)	9.80	0.002
No	3 (6)	40 (48.78)			1 (4.76)	17 (43.59)		
Smoking (%)								
Yes		61 (74.39)				26 (66.67)		
No		21 (25.61)				13 (33.33)		
History of drug use								
First use age (years)		29.59 ± 0.98				29.16 ± 1.48		
Use time (month)		52.10 ± 4.66				53.46 ± 6.54		
Daily dose (g)		0.39 ± 0.05				0.41 ± 0.06		
Drug taking mode (%)								
Snorting		80 (97.56)				39 (100)		
Injection		1 (1.22)				0 (0)		
Other		1 (1.22)				0 (0)		

Furthermore, we employed 21 healthy controls and 39 MA patients for the study of exosome miRNAs. No significant differences were found between groups in terms of age and gender (*p* > 0.05). Marital status, education and employment were significantly different between two groups. Twenty-six of patients with MA use disorder were smokers. In MA patients, MA was taken via snorting.

### Characterization of plasma exosomes

**Figure 1:**
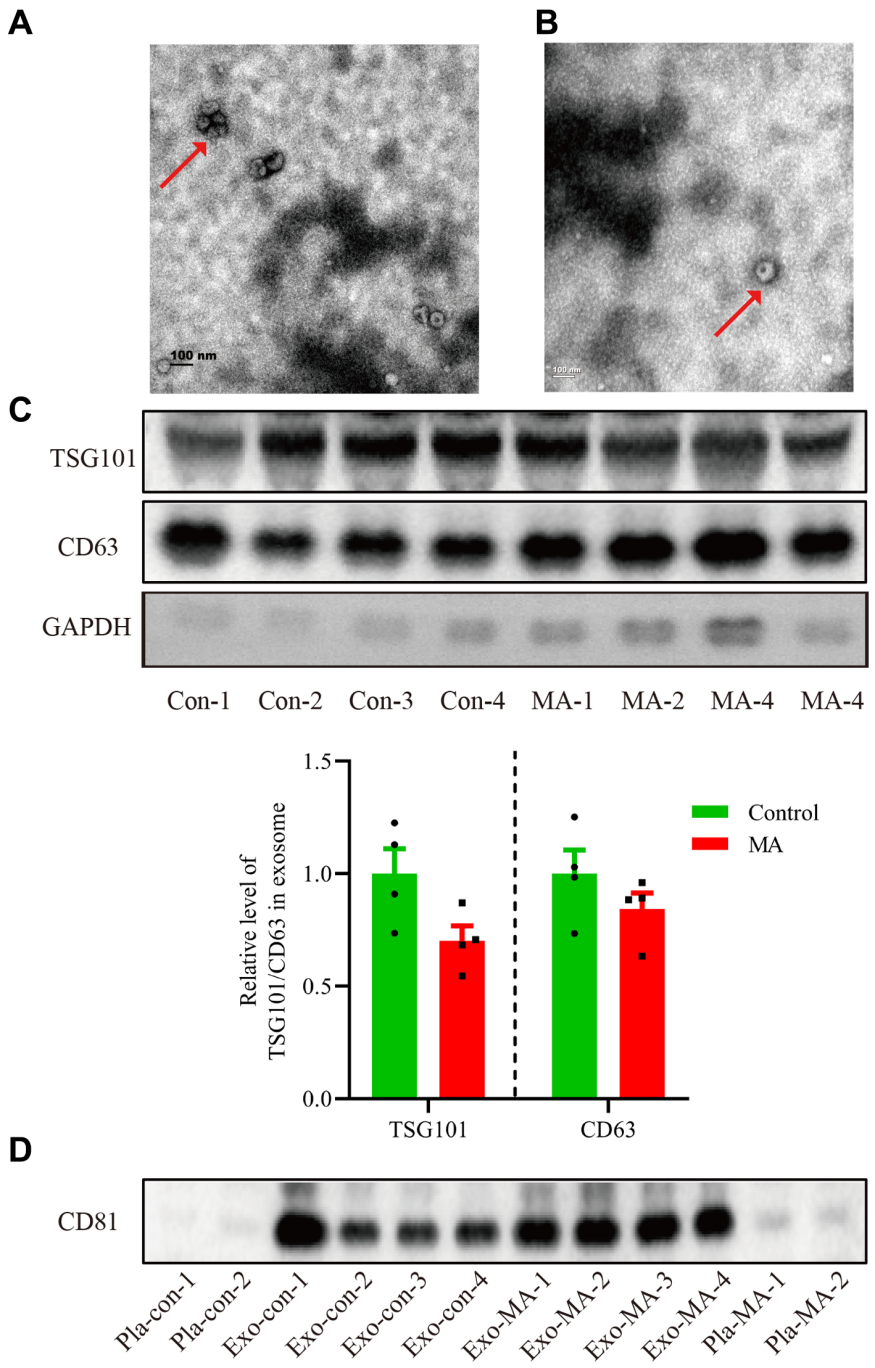
Characterization of exosomes isolated from the plasma of healthy controls and MA patients by ultracentrifugation. **(A)** Exosomes isolated from healthy controls detected by transmission electron microscopy. Scale bars: 100 nm. **(B)** Exosomes isolated from MA participants visualized by transmission electron microscopy. Scale bars: 100 nm. **(C)** Total proteins from the isolated exosomes were subjected to Western blotting analysis to confirm the presence of exosomes, using anti-CD63 and TSG101 antibodies. Histogram panel: the relative level of TSG101/CD63 protein over GAPDH. Data are expressed as mean ± SEM, *n* = 4 per group. **(D)** Total proteins from the plasma or exosome were used to perform Western blot analysis to verify the presence of exosomes, using anti-CD81 antibodies. Con, control; MA, methamphetamine; Pla, plasma; Exo, exosome.

We extracted the exosomes from the objective’s plasma according to the steps of ultracentrifugation. The morphology of exosomes was observed by transmission electron microscopy. All samples exhibited characteristic cup-shaped morphology with 30–200 nm in diameter (Figures 1A,B). To further verify the isolated exosomes, we used Western blotting analysis to identify TSG101 and CD63, the key markers for exosomes. TSG101 and CD63 were stably expressed in exosomal samples (Figure 1C). As presented in Figure 1C histogram panel, a decline trend of TSG101 expression was demonstrated in MA exosomes compared with control exosomes. However, there was no statistical significance of TSG101 expression between groups (*t* = 2.128, *p* = 0.077). Similarly, there was no significantly difference in CD63 expression between MA exosomes and control exosomes (*t* = 1.227, *p* = 0.266). Further, Western blotting analysis was used to detect the expression of CD81, an another marker for exosome. As shown in Figure 1D, CD81 was expressed in exosomal samples but not in plasma samples.

### miRNAs expression profiles between plasma and exosomes

**Figure 2:**
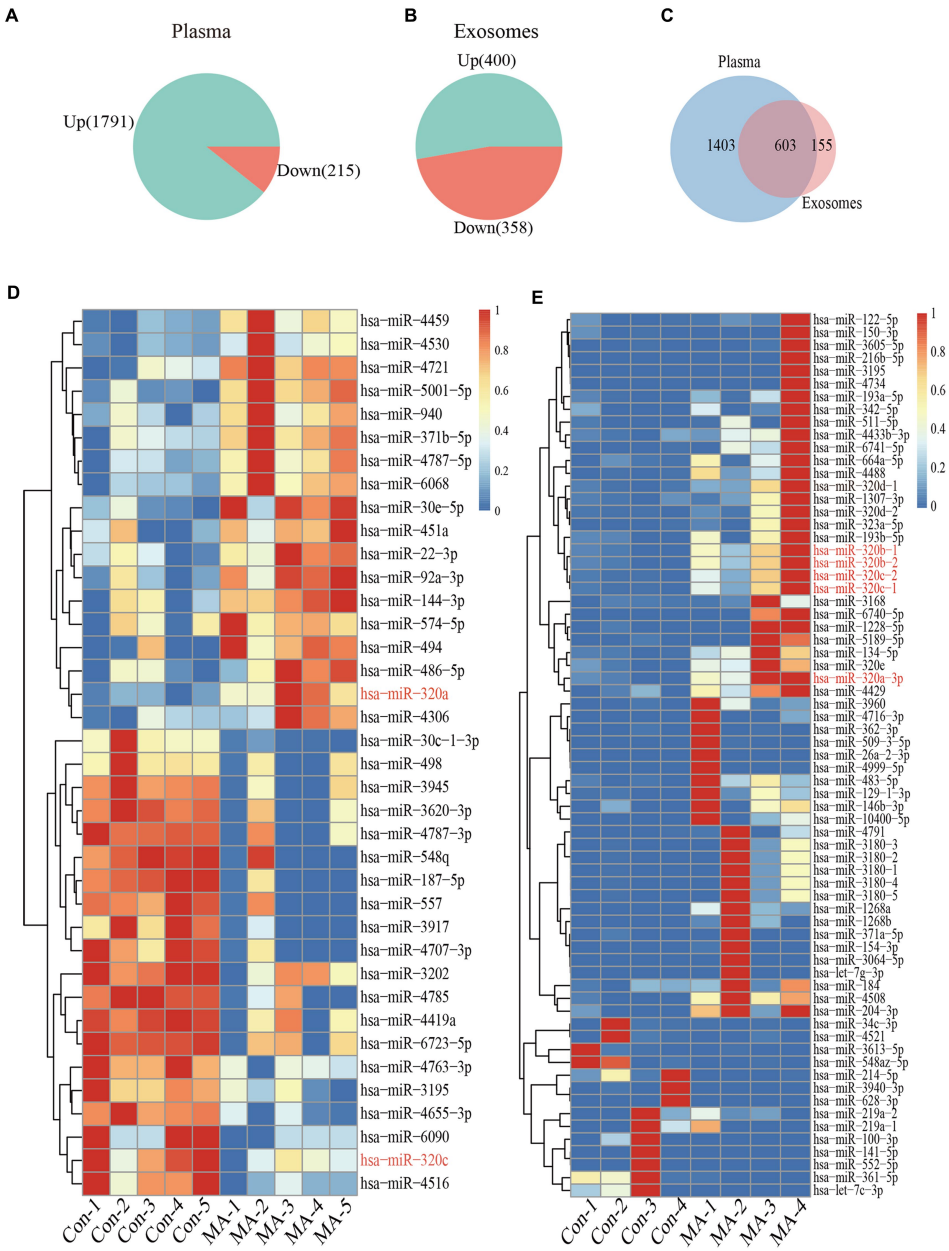
Plasma and exosomal miRNAs profiles are different in MA use disorder patients and healthy controls. Increase or decreased plasma **(A)** and exosomal **(B)** miRNAs with increases or decreases in MA use disorder patients relative to healthy controls are depicted in green or red, respectively. Venn diagrams **(C)** showed miRNAs that increased or decreased in the plasma and exosomes of subjects, and the overlap miRNAs were altered in the plasma and exosomes of subjects. Heatmap of miRNAs profiles from plasma (*n* = 5 in each group) **(D)** and exosomes (*n* = 4 in each group) **(E)** samples of MA-dependent patients and healthy control subjects. Red indicates up-regulation; blue indicates down-regulation.

miRNAs microarray analysis showed that 2006 miRNAs were expressed in plasma. 1791 miRNAs were up-regulated, and 215 miRNAs were down-regulated in MA patients compared with healthy controls (Figure 2A). Furthermore, RNA-seq revealed that 758 miRNAs were expressed in exosomes, 400 miRNAs were up-regulated, and 358 miRNAs were down-regulated in MA patients compared with healthy controls (Figure 2B). In total, 603 miRNAs were co-expressed in both plasma and exosome (Figure 2C). Microarray analysis and miRNAs sequencing identified numerous miRNAs that were differentially expressed in the plasma and exosome of MA patients and healthy controls. Total miRNAs profiles that with a mean *p* < 0.01 and fold-change >2.0 were illustrated in the heat map generated from the unsupervised clustering analysis, as shown in the heatmaps of Figures 2D,E. Interestingly, we identified two miRNAs (miR-320a-3p and miR-320c) which were differentially expressed in plasma, and five miRNAs (miR-320a-3p, miR-320b-1, miR-320b-2, miR-320c-1, and miR-320c-2), which were differentially expressed in exosomes.

### Alteration of plasma and exosome miR-320 expression in MA patients

**Figure 3:**
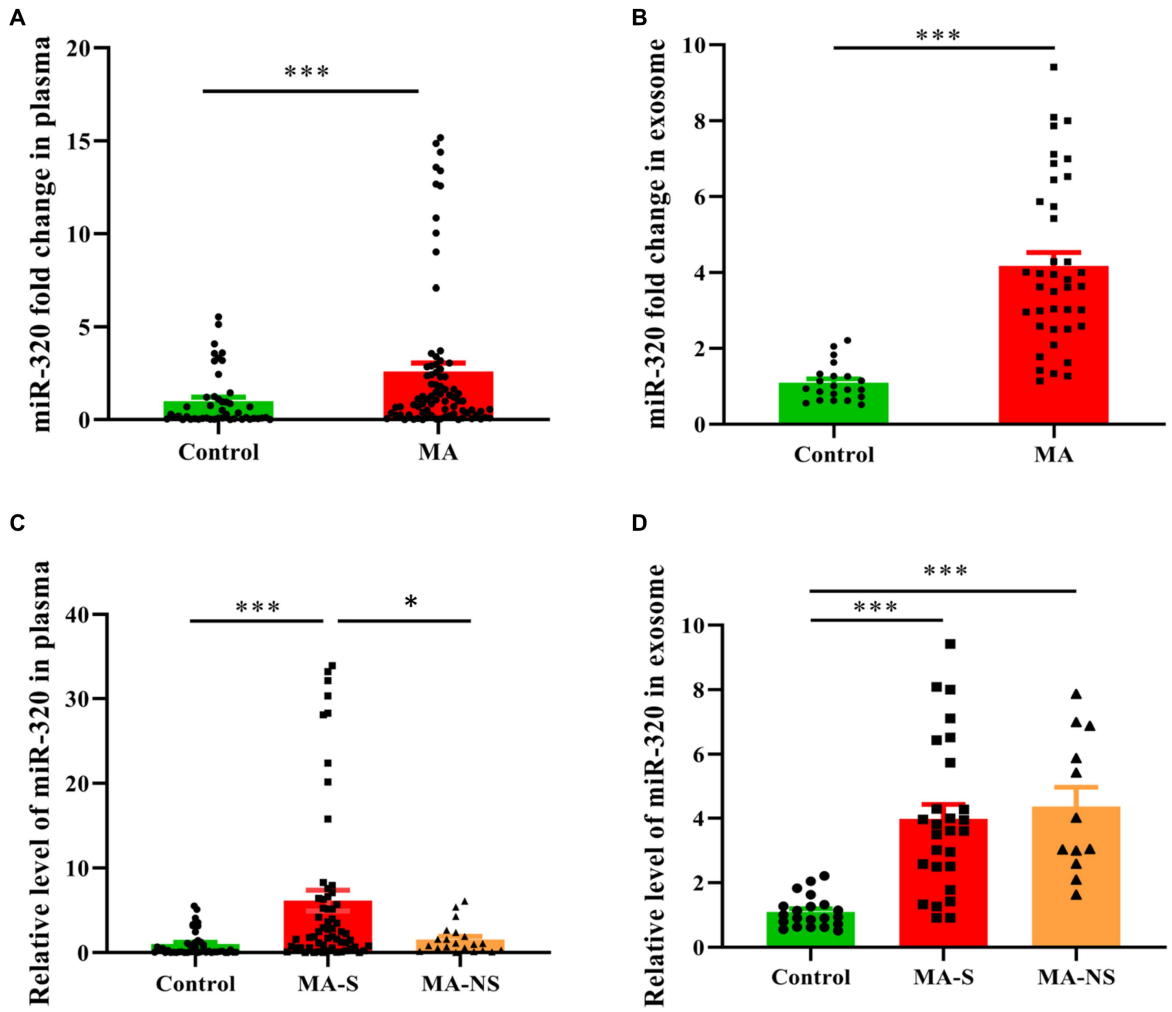
Expression of miR-320 in the plasma and exosomes from MA patients and healthy controls. **(A)** Patients with MA use disorder showed a significantly higher expression of miR-320 than healthy controls in plasma (Mann–Whitney *U*-test; *n* = 50 in control, *n* = 82 in MA use disorder, ^***^p < 0.001). **(B)** Patients with MA use disorder showed a significantly higher expression of miR-320 than healthy controls in exosomes (Mann–Whitney *U*-test; *n* = 21 in control, *n* = 39 in MA use disorder, ***p < 0.001). **(C)** Smoking significantly affected plasma miR-320 levels in MA use disorder patients (Kruskal–Wallis *H* test; *n* = 50, 61, and 21 in control, MA-S, and MA-NS groups, respectively. Con vs. MA-S, adjusted *p* < 0.001; MA-S vs. MA-NS, adjusted *p* = 0.019). **(D)** Smoking did not significantly affect plasma miR-320 in MA use disorder patients (Kruskal–Wallis *H* test; *n* = 21, 27, and 12 in control, MA-S, and MA-NS groups, respectively, Con vs. MA-S, adjusted *p* < 0.001; Con vs. MA-NS, adjusted *p* < 0.001.). Data are expressed as the mean ± SEM. **p < 0.01 ***p < 0.001.

Building on the miRNAs expression profiles in plasma and exosomes, we used RT-qPCR to validate the expression of miR-320 family (including miR-320a-3p, miR-320b, and miR-320c) in plasma and exosomes. Cel-miR-39 and miR-423-5p were determined as the internal references to identify miRNAs in plasma and exosomes. The expression of miR-320 family was significantly higher in the plasma from MA patients than that from healthy controls (*U* = 1025.50, *p* < 0.001, Figure 3A). Similarly, the expression of miR-320 family was significantly higher in the exosomes from MA patients than those from healthy controls (*U* = 44.00, *p* < 0.001, Figure 3B). Both MA and tobacco are often used together in MA patients. Therefore, we distinguished the expression of miR-320 in plasma between smoker and nonsmoker groups in MA patients (smokers and nonsmokers denoted as MA-S and MA-NS, respectively) and compared with healthy controls. The significant effects were found between groups on plasma miR-320 expression (*H* = 30.35, *p* < 0.001), and *post hoc* comparisons showed the increased expression of plasma miR-320 in MA-S (adjusted *p* < 0.001) and the decreased expression of plasma miR-320 in MA-NS (adjusted *p* = 0.019). There were no significant differences in the expression of plasma miR-320 between healthy controls and MA-NS (adjusted *p =* 0.546, Figure 3C). Additionally, we analyzed the expression of miR-320 in exosomes based on cigarette smoking status in MA patients, Kruskal–Wallis *H* test showed the significant effects between MA-S and MA-NS groups on exosomal miR-320 expression (*H* = 32.37, *p* < 0.001), and *post hoc* comparisons showed the increased expression of exosomal miR-320 in MA-S and MA-NS (adjusted all *p* < 0.001) compared to the control group, respectively. Moreover, cigarette smoking failed significantly to alter exosomal miR-320 expression between MA-S and MA-NS groups (adjusted *p* = 0.60, Figure 3D).

### Diagnostic accuracy of miR-320 panels as diagnostic biomarkers

**Figure 4:**
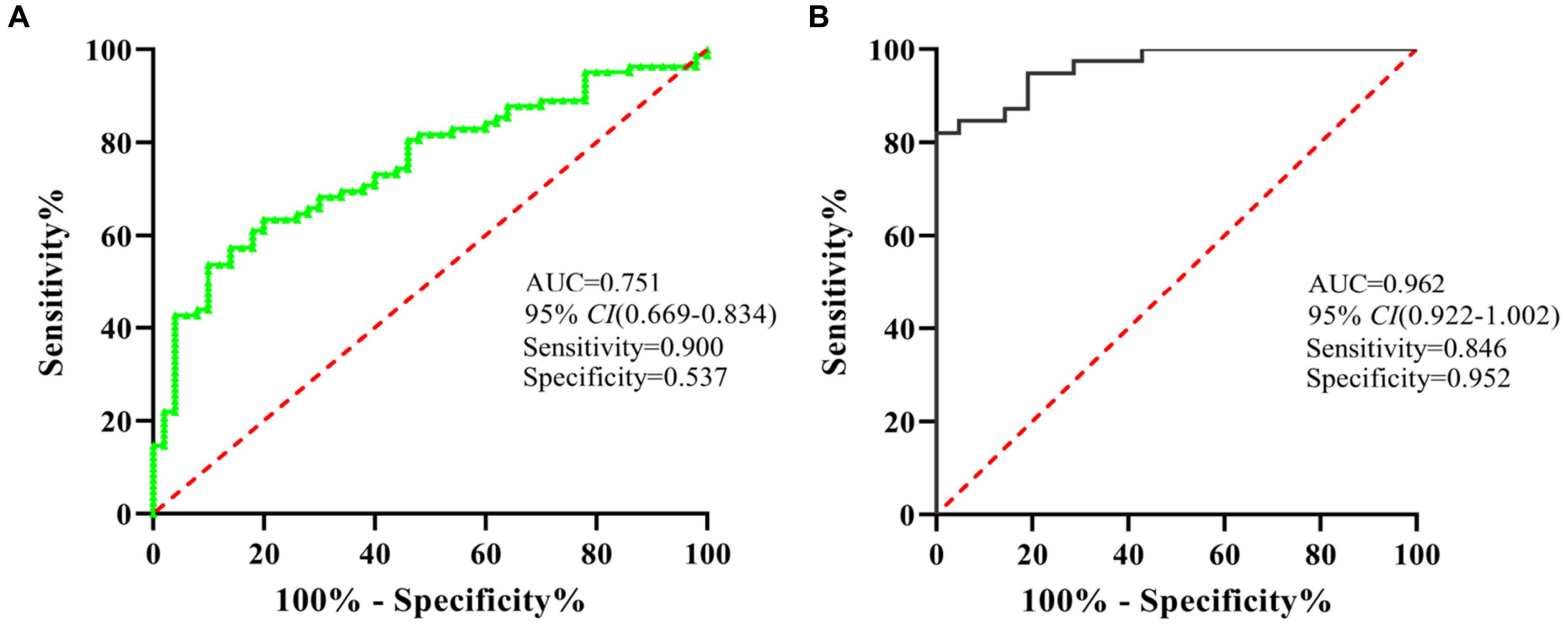
ROC analysis showed that miR-320 had a decent diagnostic performance for the identification of MA use disorder patients. **(A)** ROC curve analysis of miR-320 in plasma. **(B)** ROC curve analysis of miR-320 in exosomes. CI, confidence interval.

To evaluate the diagnostic value of miR-320 for MA use disorder, we applied the ROC curve. Subsequent analysis of diagnostic power showed that miR-320 in the exosomes for MA use disorder was significantly greater than that in plasma (AUC values of 0.962 and 0.751, respectively, with a sensitivity and specificity in exosomes of 0.867 and 0.551, respectively) (Figures 4A,B). Although exosomes derived miRNAs are part of total miRNAs in plasma, our results suggested that the miR-320 isolated from plasma exosomes was higher AUC value for diagnosis of MA use disorder than the miR-320 directly isolated from plasma.

### Clinical significance of miR-320 in MA use disorder

As shown in Table 2, several variables were found to be statistically different between two groups. And these factors might have influences on the expression of miR-320. Logistic regression analysis was performed to explore the expression of miR-320 by adjusting confounding factors. miR-320 expression in MA patient was significantly higher than that in healthy controls (adjusted odds ratio (AOR) = 0.67, 95%*CI* = 0.53–0.86, *p* = 0.002; adjusted odds ratio (AOR) = 0.02, 95%*CI* = 0.002–0.286, *p* = 0.004) in plasma and exosome, respectively (Table 3).

**Table 3:** Variables in logistic regression analysis.

Variables	Plasma (Control vs. MA use disorder)			Exosome (Control vs. MA use disorder)		
	*β*	OR (95%CI)	*p*	*β*	OR (95%CI)	*p*
Age (years)	−0.13	0.88 (0.81–0.95)	<0.001			
Education						
Primary and below		1.00 (Reference)			1.00 (Reference)	
Middle school	−0.21	0.82 (0.04–16.35)	0.89	−1.87	0.16 (0.001–19.32)	0.452
High school	−3.14	0.04 (0.01–0.51)	0.013	−3.67	0.03 (0.00–5.02)	0.174
College and above	−5.95	0.003 (0.00–0.04)	<0.001	−8.76	0.00 (0.00–0.22)	0.018
Employment		1.00 (Reference)				
Yes	0.153	1.17 (0.999–1.359)	0.051			
No						
miR-320	−0.40	0.67 (0.53–0.86)	0.002^a^	−3.865	0.021 (0.002–0.286)	0.004^b^

OR, odds ratio; CI, confidence interval. ^a^Adjusted for age, marital status, education, employment; ^b^Adjusted for marital status, education, employment.

We further explored the correlation between clinical characteristics and miR-320 expression in MA patients. As shown in Table 4, plasma miR-320 expression was positively associated with cigarette smoking, age of onset, and daily dose of MA use, indicating the potential of miR-320 as a biomarker for the diagnosis of MA use disorder.

**Table 4:** Correlation analysis between miR-320 expression level in MA disorder group.

Variables	Plasma		Exosome	
	*r*_s_	*p*	*r*_s_	*p*
Smoking	0.335	0.012	−0.135	0.411
Age of onset (years)	0.24	0.03	0.064	0.699
Use time (month)	−0.045	0.688	0.203	0.216
Daily dose (g)	0.292	0.008	−0.112	0.496
Drug taking mode	0.206	0.063	-	-
Frequency of use	−0.078	0.485	0.096	0.562
Withdrawal (months)	0.097	0.384	−0.244	0.135

### Targets pathways of miR-320 and protein–protein interaction (PPI) network

**Figure 5:**
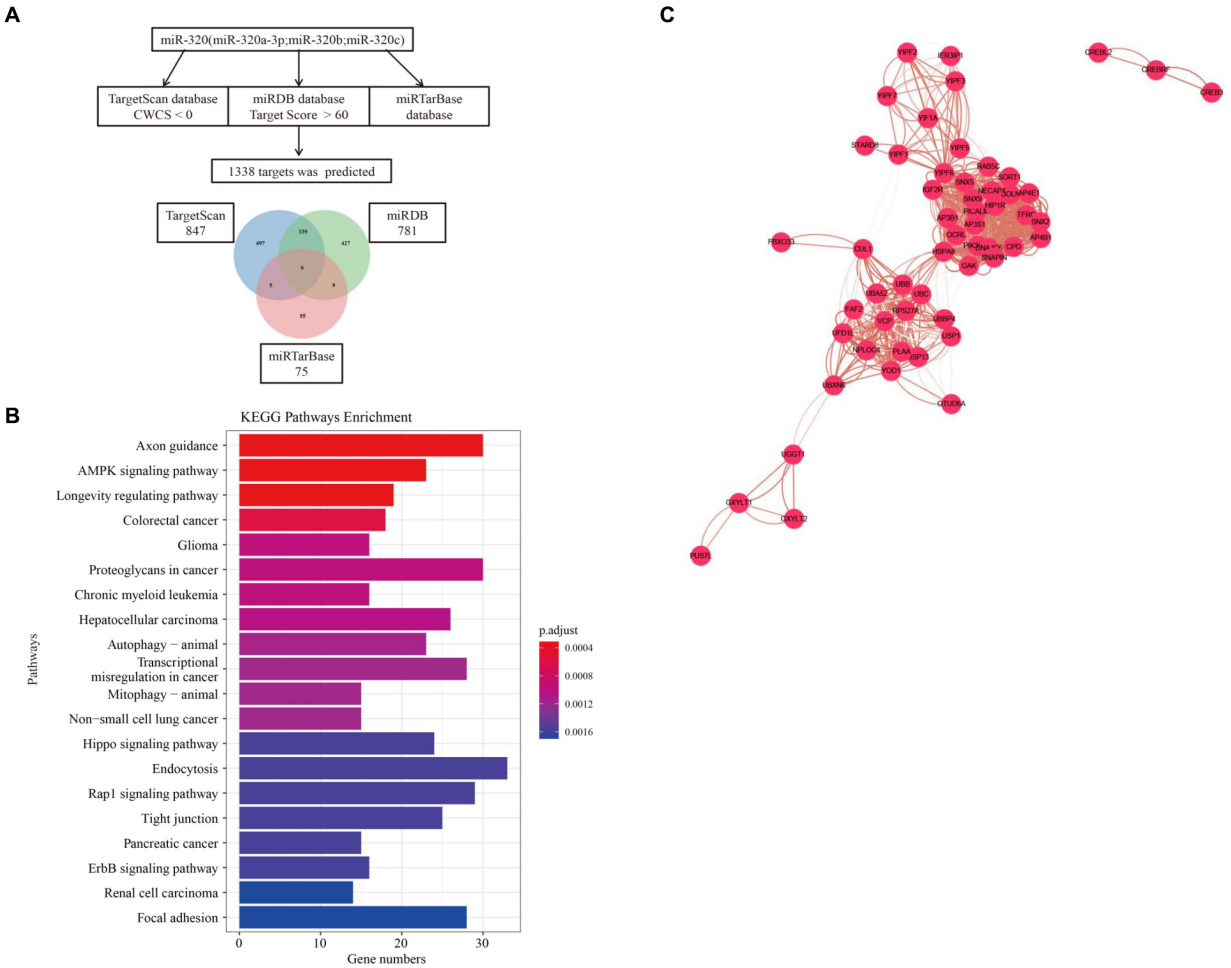
Target and pathway prediction for miR-320. **(A)** miRNAs target prediction workflow using miR-320a-3p, miR-320b, and miR-320c. **(B)** Top 20 canonical pathways associated with the predicted mRNA targets. **(C)** The PPI network of overlaped genes was generated using STRING and visualized with Cytoscape. PPI, protein-protein interaction.

We used three online bioinformatic prediction databases, TargetScan,^1^ miRDB^2^ and miRTarBase^3^ to screen potential targets of miR-320. We identified 1,967 predicted targets for miR-320 (including miR-320a-3p, miR-320b, and miR-320c). Basing on the inclusion criteria, a total of 1,338 targets were used for subsequent analysis. The TargetScan database predicted 847 targets of miR-320, whereas the miRDB and miRTarBase database predicted 781 and 75 targets of miR-320, respectively. The three target lists overlapped 6 target genes (CREBRF, FBXO33, GXYLT1, PCDHA2, YIPF6, YOD1) for a total of 979 unique targets in the relationship among TargetScan, miRDB and miRTarBase (Figure 5A). KEGG pathway enrichment analyses on these predicted genes identified 61 canonical pathways. The canonical pathways were associated with cardiovascular disease, synaptic plasticity, and neuroinflammation, which are associated with MA dependency (Figure 5B). To identify the shared genes signatures in three databases in MA use disorder, we further constructed the PPI network at protein levels by using the STRING^4^ and visualized with Cytoscape (version: 3.6.1). As shown in Figure 5C, in which contained 1,350 nodes and 8,007 edges. Functional enrichment analysis showed the functions of genes of PPI network were mainly associated with regulation of receptor localization to synapse, nervous system development and γ-aminobutyric acid (GABA) receptor binding. Thirty-three genes were considered as hub genes owing to high degrees (degree >10) in the network. Top 10 hub genes were YIPF6, HSPA8, GAK, RAB5C, AP4B1, AP3B1, CPD, HIP1R, SNX5, and SNX9.

## Discussion

In this study, the expression of miR-320 family significantly increased in the plasma and exosomes of patients with MA use disorders, indicating that miR-320 might be used as a blood-based biomarker for the diagnosis of MA use disorder. In addition, the expression of plasma miR-320 was related to the cigarette smoking, age of onset, and daily dose of MA use in patients with MA use disorders. Furthermore, ROC curve analysis showed that exosomal miR-320 was more effective than plasma miR-320 in the diagnosis of MA use disorder.

Previous studies have shown that plasma and exosomal miRNAs play important roles by regulating gene expression on the pathophysiology of drug addiction (14, 15). The use of MA can alter gene expression in key brain regions, suggesting that importance of miRNAs in MA addiction. The expression of miR-181a, miR-15b, let-7e, and let-7d decreased in the plasma of MA patients compared with those in the plasma of healthy controls, oppositely, the expression of miR-9-3p significantly increased in the serum of MA-dependent patients, and miR-9-3p might be used as a biomarker for the diagnosis of MA addiction (16, 17). Furthermore, serum exosomal and hippocampal miRNAs levels were significantly higher in rats with MA than those in control rats, KEGG analysis showed that the potential targets pathway of the above-mentioned miRNAs was the regulation of glutamatergic synaptic plasticity (18). In addition, miR-29a-3p expression increased in circulating plasma EVs of MA patients, suggesting that miR-29a-3p might serve as a diagnostic biomarker for MA use disorder (19). Another study found that miR-137 significantly reduced in plasma EVs of patients with MA abstinence, and indicated that miR-137 can be used as a potential biomarker for MA withdrawal syndrome (20). Recently, 169 miRNAs were found to be altered in the plasma EVs of patients with MA use disorders, and demonstrated that the expression of miR-301a-3p, miR-382-5p, and miR-628 were related to MA exposure and the age of first drug use (21). We have performed bioinformatic analysis of miRNAs with diagnostic potential reported in the above studies, and found that the downstream signaling molecules and neurotransmitters regulated by these miRNAs were related with glutamatergic synaptic plasticity, ATP binding and neuroinflammatory pathways. Therefore, further exploration and discovery of specifically expressed circulating plasma and exosomal miRNAs as biomarkers for the diagnosis and treatment of MA addiction has great clinical value.

miR-320 family (including miR-320a-3p, miR320b, and miR-320c) are located on chromosome 8. Our previous study suggested that the expression of miR-320a-3p significantly increased in heroin-dependent patients compared with healthy controls, and suggested that miR-320a-3p might be used as a biomarker for the diagnosis of heroin addiction (22). Up to now, miR-320a-3p has not been reported in methamphetamine, cocaine, or other drug addictions. Interestingly, the dysfunction of miR-320a-3p was closely related to Alzheimer’s disease and a variety of central nervous system diseases (23–26). Moreover, circulating miR-320a-3p exhibited dysregulation in psychiatric disorders and has been shown to act as a diagnostic biomarker. For instance, plasma miR-320a-3p was significantly down-regulated in patients with major depression (23). Meanwhile, miR-320a was down-regulated in the plasma of autism patients (27). Interestingly, circulating miR-320a-3p, miR-320b, and miR-320c were significantly up-regulated in patients with Alzheimer’s disease or mild cognitive impairments, and these miRNAs exhibited strong diagnostic abilities for Alzheimer’s disease (26). Raheja et al. identified six miRNAs (including miR-320a, miR-320b, and miR-320c) which were down-regulated in amyotrophic lateral sclerosis (ALS) patients compared with healthy controls (28). Moreover, the abnormal expression of circulating miR-320b was related to schizophrenia (25). Notably, repeated MA use affects vasoconstriction, pulmonary hypertension, atherosclerotic plaque formation, cardiomyopathy, and pathological phenotypes, similarly as common neurodegenerative disorders (29, 30). Coincidentally, miR-320a-3p was found to be significantly elevated in the plasma of paroxysmal atrial fibrillation patients compared with healthy controls. Furthermore, miR-320a-3p plasma levels were positively correlated with CHA2DS2-Vasc score and were elevated in subjects with CHA2DS2-Vasc ≥2 (31). In addition, circulating miR-320b represents a biomarker for atherosclerosis (32). Based on the above studies, we speculated that the dysfunction of miR-320 in plasma and exosomes could reflect the corresponding dysfunction in the brain caused by MA addiction.

Previously, our group have found that the levels of miR-181a, miR-320a, and let-7b-5p in circulating plasma significantly increased in heroin-dependent patients, and demonstrated that these miRNAs can distinguish heroin addicts from healthy controls (22, 33). Tobacco affects the central and circulating expression of miRNAs and is prevalent in drug-addicted patients (21, 34). A previous study showed let-7d and miR-320 were upregulated in the smoking population, indicating smoking may be a confounding factor when using the expression of serum miRNAs for diagnosing pathological conditions. In this study, we observed that the expression of miR-320 significantly increased in the plasma of MA-S compared with that in MA-NS. However, in a recent study, the expression of miR-137 in circulating EVs did not change in MA use disorder with tobacco exposure (20). Our results that exosomal miR-320 is not differentially expressed between MA-S and MA-NS were consistent with their findings. These results suggested that tobacco might be an important factor leading to the differential expression profiles of plasma miRNAs in MA use disorder, and its use may affect plasma and exosomal miRNAs through different mechanisms. Furthermore, we analyzed the relationship between the expression of miR-320 and the clinical characteristics of MA patients, and found that the increased plasma miR-320 expression in MA patients was positively correlated with cigarette smoking, age of onset, and daily dose of MA use, and negatively correlated with occupational status. It was previously reported that the decreased expression levels of miR-181a, miR-15b, let-7e, and let-7d in the plasma of patients with MA use disorder were negatively correlated with the frequency of MA use in the past 15 days (16). However, in our study, the expression of miR-320 was not correlate with the frequency of MA use, which may be attributable to the time window when blood samples were collected from patients from patients during the withdrawal period, and to different expression profiles of miRNAs shown in different addiction stages. In other studies, the expression level of miR-9-3p was increased in the serum of patients with MA addiction (17). Our previous has shown that the elevated expression of miR-181a was positively correlated with the frequency of heroin-dependent use, and negatively correlated with the daily dosage in patients with heroin use disorder (33). However, in this study, the expression of miR-320 was positively correlated with MA daily use, which may be due to different illicit drugs generate different miRNAs expression profiles, and the specific dysregulated miRNAs molecules.

Patients with MA abstinence exhibited a significant reduction in the circulating EVs miR-137 (20). Moreover, 88 miRNAs had changed (by at least 1.2-fold), 107 miRNAs were found to be altered in exosomes in smoking and non-smoking MA-dependent patients (by at least 1.2-fold). Based on the cumulative criteria of a 1.2-fold change in expression, an effect size of 0.8, and AUC ≥ 0.75, 8 miRNAs were identified in MA patients, as well as 15 smoker miRNAs of interest out of the top ranked miRNAs, miR-301a-3p and miR-382-5p overlapped between MA-dependent patients and smokers. Furthermore, the authors analyzed the relationship between the differentially expressed miRNAs in the exosomes of patients with MA use variables, and found that miR-301a-3p, miR-382-5p, and miR-628-5p were related to the age of onset and percentage of lifetime using MA. Finally, the three miRNAs associated with lifetime exposure to MA (miR-301a-3p, miR-382-5p, and miR-628) were used for mRNA target prediction with the online analysis tools (21). In our study, we also analyzed the relationship between exosomal miRNAs of MA patients and MA exposure. Unfortunately, we did not find the relationship between the expression of miR-320 in the exosomes and the characteristics of MA patients. The study was not to detect the potential cellular and molecular targets of circulating exosomes during MA addiction and to specifically isolate exosomes in plasma. Many bodily cell types and biological fluids, including blood, saliva, urine, breast milk, and cerebrospinal fluid, have been shown to release exosomes (35). MA use disorder is a chronic recurrent encephalopathy, exosomes from brain tissue sources of MA addicts may contribute more to its pathophysiology, particularly, exosomes from brain tissue are essential for the development of MA addiction. Notably, in this study, the exosomes isolated from peripheral plasma, and the expression changes of miR-320 in circulating plasma and exosomes may not actually reflect the abnormal expression of miR-320 in the brain. Therefore, future research may require isolation and analysis of exosomes from the brain, cerebrospinal fluid, and tracking of their potential targets.

Our study also had several limitations. The small sample size in the study precludes us from asserting circulating miR-320 to be a diagnostic biomarker in MA use disorder. Moreover, there was a considerable gender difference between MA and control groups. Females are more sensitive to the effects of MA, which may lead to differential expression profiles of miRNAs (36–38). Consistently, we found that tendency that expression levels of exosomal miR-320 in females MA patients were with higher than that in males. We should expand the sample size and verify the specificity of miR-320 expression in MA addicts in future studies. In addition, smoking, depression, fantasy, and antidepressant treatments are common confounding factors in MA user. In our study, we only analyzed the relationship between smoking and the expression of miR-320 in MA patients, and the potential interactions among these confounding factors should be investigated in the future. Furthermore, we predicted that the target gene of miR-320 include CREB that related to MA addiction, suggested that the biological function of the specific genetic networks or targets regulated by miR-320 family in MA addiction should be investigated in animal models in the future. Finally, considering that MA use disorders are complex and often accompanied by neuropsychiatric disorders. Although miR-320 could be distinctly increased in MA use disorder patients, the more work need to differentiate MA use disorder from other neuropsychiatric disorders in clinical practice. In future research, more miRNAs specifically expressed in MA patients should be mined to jointly diagnose MA addiction.

In conclusion, the expression of miR-320 family significantly increased in the plasma and exosomes of MA addictor in the present study, indicating that miR-320 family in the circulating plasma or exosomes hold diagnostic potential as a blood-based marker of MA use disorder. Furthermore, exosomal miR-320 might be more effective biomarker than plasma miR-320 in the diagnosis of MA use disorder.

## Data availability statement

The datasets presented in this study can be found in online repositories. The names of the repository/repositories and accession number(s) can be found at: https://www.ncbi.nlm.nih.gov/geo/query/acc.cgi?acc=GSE224658 (GSE224658) and https://www.ncbi.nlm.nih.gov/geo/query/acc.cgi?acc=GSE224657 (GSE224657).

## Ethics statement

The studies involving human participants were reviewed and approved by the Ethics Committee of Ningbo Kangning Hospital (No. NBKNYY-2020-LC-53). The patients/participants provided their written informed consent to participate in this study. Written informed consent was obtained from the individual(s) for the publication of any potentially identifiable images or data included in this article.

## Author contributions

WX drafted the manuscript. QH, YZ, LL, XC, WC, and DZ undertook the recruitment of patients and completed the experiments. WX, MW, XX, and ML provided statistical advice on the study design and analyzed the data. WZ and HL conceived the study and its design. All authors read and approved the final manuscript.

## Funding

This work was supported by the Medical Health Science and Technology Project of Zhejiang Provincial Health Commission (grant number 2021KY1065, 2022KY1175, 2023KY296), the Natural Science Foundation of Ningbo (grant number 2021J273), Innovation Project of Distinguished Medical Team in Ningbo (grant number 2022030410) and Zhejiang Medical & Health Leading Academic Discipline Project (grant number 00-F06).

## Conflict of interest

The authors declare that the research was conducted in the absence of any commercial or financial relationships that could be construed as a potential conflict of interest.

## Publisher’s note

All claims expressed in this article are solely those of the authors and do not necessarily represent those of their affiliated organizations, or those of the publisher, the editors and the reviewers. Any product that may be evaluated in this article, or claim that may be made by its manufacturer, is not guaranteed or endorsed by the publisher.

## Footnotes

^1^ https://www.targetscan.org

^2^ https://mirdb.org

^3^ http://mirtarbase.mbc.nctu.edu.tw/index.html

^4^ http://string-db.org
